# Invasive Disease Due to *Neisseria meningitidis*: Surveillance and Trends in Israel Prior to and during the COVID-19 Pandemic

**DOI:** 10.3390/microorganisms11092212

**Published:** 2023-08-31

**Authors:** Chen Stein-Zamir, Hanna Shoob, Nitza Abramson, Lea Valinsky, Joseph Jaffe, David Maimoun, Sharon Amit, Maya Davidovich-Cohen

**Affiliations:** 1 Jerusalem District Health Office, Ministry of Health, 86 Jaffa Road, Jerusalem 9434124, Israel; hanna.shoob@lbjr.health.gov.il (H.S.); nitza.abramson@lbjr.health.gov.il (N.A.); 2 Faculty of Medicine, Hadassah Braun School of Public Health, The Hebrew University of Jerusalem, Jerusalem 9112102, Israel; 3 Public Health Laboratories Jerusalem, Public Health Services, Ministry of Health, Jerusalem 9546208, Israeljoseph.jaffe@moh.gov.il (J.J.); david.maimoun@moh.gov.il (D.M.); maya.davidovich@moh.gov.il (M.D.-C.); 4 Clinical Microbiology, Sheba Medical Center, Ramat Gan 5266202, Israel; sharon.amit@sheba.health.gov.il

**Keywords:** *Neisseria meningitidis*, invasive meningococcal disease, molecular epidemiology, clones

## Abstract

Invasive meningococcal disease (IMD) is a devastating disease with significant mortality and long-term morbidity. The COVID-19 pandemic and containment measures have affected the epidemiology of infectious pathogens. This study’s aim was to assess IMD trends in Israel prior to and during the COVID-19 pandemic. The *Neisseria meningitidis* invasive infection is a notifiable disease in Israel. Laboratory analysis includes serogrouping and molecular characterization. The overall national IMD incidence rate (1998–2022) was 0.8/100,000 population. The IMD incidence rates declined during the pandemic years (0.3/100,000 in 2020–2022 vs. 0.9/100,000 in 1998–2019). The number of notified IMD cases declined by 65% in 2020–2022. The case fatality rate among laboratory-confirmed IMD cases was 9% (47/521, 2007–2022). Mortality risk markers included cases’ age (older) and socio-economic status (lower). Overall, most *Neisseria meningitidis* isolates were of serogroup B (62.6%), and the most prevalent clonal complex (CC) was CC32 (24.2%). Serogroup B prevailed in cases aged 0–9 years (74.5%) and less in cases aged 10 years and above (39%). *Neisseria meningitidis* serogroups and CC distribution altered recently with a decline in serogroup B fraction, an increase in serogroup Y, and a decline in CC32. Ongoing IMD surveillance is necessary to assess trends in circulating strains and support decision-making on meningococcal vaccination programs.

## 1. Introduction

*Neisseria meningitidis* (*N. meningitidis*) is a Gram-negative bacterium-causing invasive disease. An invasive meningococcal disease (IMD) is a life-threatening and devastating disease associated with significant morbidity and mortality as well as long-term sequelae and considerable disability among survivors [1]. The main clinical presentations of IMD include meningococcal meningitis and meningococcal sepsis (meningococcemia) [1]. Globally, six *N. meningitidis* serogroups (A, B, C, W, X, and Y) account for almost all IMD cases. IMD is a major public health challenge; the *N. meningitidis* serogroups distribution and the IMD incidence rates vary markedly between geographical regions and periods [2]. IMD mortality varies by *N. meningitidis* serogroup, geography, and age group [3]. A systematic review and meta-analysis (some 40 studies from the European Region, Eastern Mediterranean Region, Western Pacific Region, and the Region of the Americas; 163,758 cases) assessed IMD case fatality rates (CFR) of 4.1% to 20.0%, and a pooled CFR of 8.3% [3]. Serogroup B cases showed lower CFR (6.9%) compared to W (12.8%), C (12.0%), and Y (10.8%); serogroup A cases were not included. CFR in laboratory-confirmed cases were 9.0% in infants, 7.0% in children, 15.0% in young adults, and 15–20% in older adults [3].

In 2020, the World Health Organization endorsed a global road map that sets forth a vision to defeat meningitis by 2030. The road map sets three goals towards a “world free of meningitis”, including the elimination of bacterial meningitis epidemics, reduction of vaccine-preventable bacterial meningitis cases by 50% and deaths by 70%, and reduction of disability and improvement of quality of life among survivors [4,5]. Lessons learned from the COVID-19 pandemic indicate that global cooperation is essential for improving surveillance, diagnosis, and prevention of a global public health threat [5].

Previous epidemiological studies in Israel have estimated the overall IMD annual incidence, as approximately one per 100,000 population, during the last two decades differing between age groups [6,7,8]. The IMD incidence rates were higher among children and specifically in infants and toddlers (accounting for some two-thirds of the cases), with young children residing in low socio-economic areas showing higher incidence rates [6,7,8]. According to the bacterial isolates of *N. meningitidis*, the leading serogroup in Israel has been serogroup B (MENB) [6,7,8].

Invasive meningococcal infections attributable to the *N. meningitidis* serogroups A, B, C, W-135, and Y are potentially preventable by commercially available vaccines [7,8,9]. In Israel, three conjugate meningococcal vaccines (A-C-W-135-Y) and two vaccines against serogroup B (MenB-4C vaccine, Bexsero™, and MenB-fHbp vaccine, TruMenBa™) are registered [9]. To date, the national routine immunization program does not include universal meningococcal vaccinations, and the recommendations relate principally to risk factors; while recommendations for healthy children for MENB vaccination exist, the vaccine is not provided in the national routine program [9]. 

The global COVID-19 pandemic and the utilization of containment measures have affected the epidemiological patterns of multiple infectious pathogens. A recent study assessed the incidence of invasive disease caused by *Streptococcus pneumoniae*, *Haemophilus influenzae*, and *N. meningitidis*, during the COVID-19 pandemic, in 26 countries and territories [10]. The Invasive Respiratory Infection Surveillance Initiative data showed a reduction in invasive diseases due to these three pathogens in early 2020, corresponding to the widespread introduction of COVID-19 containment measures [10]. Comparable trends showing the incidence of decline in case notifications of invasive *N. meningitidis* infections during the COVID-19 pandemic have been reported in the Netherlands and in France [11,12,13]. 

The aim of the current study was to investigate the evolving epidemiology of invasive *N. meningitidis* infections (before and during the COVID-19 pandemic years) in Israel through the assessment of the population-based epidemiological data and the characteristics and genomics of *N. meningitidis* bacterial isolates.

## 2. Methods

### 2.1. Epidemiological Methods

Invasive infection caused by *N. meningitidis* is a notifiable disease by law in Israel. Physicians and microbiological laboratories are required to notify IMD cases promptly to the District Health Office and to send the bacterial isolate and/or DNA to the National Reference Molecular Laboratory. The district health teams perform epidemiological investigations on notified IMD cases using a structured questionnaire with additional information collected from community clinics and hospital records, as applicable. The health teams provide post-exposure prophylaxis, control measures, and guidance to families and communities [8]. Case definition and ascertainment are performed according to the national guidelines. Case confirmation requires the isolation of *N. meningitidis* from blood, cerebrospinal fluid (CSF), or other sterile sites in a patient with a clinically compatible illness [8]. 

We assembled the IMD incidence data for the calendar years 1998–2022 and the laboratory and epidemiological data for the calendar years 2007–2022. The variables included were age, gender, date of IMD onset, and the socio-economic (SE) rank of residence locality. An ordinal scale of localities from rank 1 (lowest) to 10 (highest) determined the SE rank [14]. We retrieved the IMD case notifications from the weekly epidemiological reports issued by the Ministry of Health [15] and calculated IMD incidence rates based on notification data. Israel’s population was 9.37 million in 2021; children under 15 comprised 28% of the national population. The IMD cases were appraised according to six age groups: infants <1 year (population of 185,000), toddlers aged 1–4 years (730,200), children aged 5–9 years (852,950), adolescents aged 10–17 years (1,296,500), persons aged 18–44 years (3,377,000) and persons ≥45 years of age (2,929,800) [16]. Regarding ethnicity, 79% of the national population are Jews and others, and 21% are Arabs [16].

### 2.2. Laboratory Methods

According to the Ministry of Health guidelines, the microbiological laboratories routinely submit all *N. meningitidis* isolates to the National Reference Center for Meningococci of the Ministry of Health for verification, bacterial serogrouping, and antimicrobial susceptibility testing. The laboratories also send *N. meningitidis* isolates or DNA (PCR cases confirmation without a bacterial isolate) to the National Molecular Laboratory of the Ministry of Health.

The molecular characterization included multi-locus sequence typing (MLST) genotyping (since 2007, *n* = 462) and whole-genome sequencing (WGS) (since 2017, n = 142). The National Molecular Laboratory performs MLST genotyping according to the standard *N. meningitidis* protocol (available at http://pubmlst.org/neisseria/info, accessed on 30 May 2023). The allele number, sequence type (ST) and clonal complex (CC) were determined in the *Neisseria* MLST database. For WGS analysis, genomic DNA was extracted using the QiaSymphony platform (QIAGEN) and quantified with the Qubit fluorimetry 1.0 ng of DNA used for Illumina Nextera XT library preparation. Short sequence reads were generated on HiSeq (Illumina, San Diego, CA, USA), aiming at >100× coverage. The laboratory used the Linux distribution Ubuntu 22.04.1 to run the workflow. Initially, we checked the reads of the quality control with the FastQC v0.11.9, cleaned them up, and trimmed them using Trimmomatic v0.39. The assembly was performed by the Spades assembler v3.13.1. We utilized Python 3.8.13 to check the serogroups, and all the typing—the MLST, Bexsero antigen sequence types, fine-typing antigens—and the meningococcal deduced vaccine antigen reactivity (MenDeVAR), was carried out using the PubMLST database [17].

### 2.3. Statistical Analysis

Statistical analysis was performed with IBM SPSS Statistics (Version 28.0. IBM Corp.: Armonk, NY, USA). We compared the IMD proportions according to the variables by odds ratio (OR) or rate ratio (RR) with 95% confidence intervals (95%CI). We used the Pearson chi-square test for comparing categorical variables and the *t*-test for continuous variables. We calculated *p*-values using the Fisher exact test. A multiple regression model was implemented for risk markers for IMD mortality. A *p*-value of <0.05 was considered significant for all comparisons.

The study was approved by the Ministry of Health, Israel, and performed in accordance with the relevant Ministry of Health national guidelines.

## 3. Results

### 3.1. Invasive Meningococcal Disease Incidence Trends 

**Figure 1:**
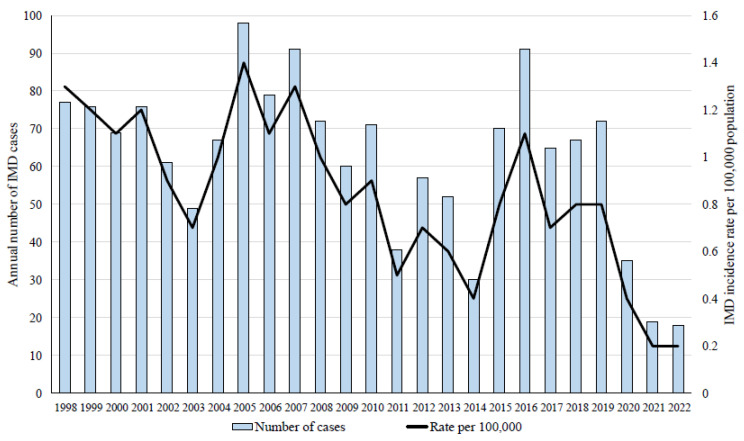
The annual number of notified invasive meningococcal disease (IMD) cases and the annual incidence rates of IMD per 100,000 population during the years 1998–2022 in Israel.

During 1998–2022, 1560 cases of IMD have been notified in Israel with an overall incidence rate of 0.8/100,000 population. The IMD incidence rates varied considerably between the years (Figure 1). We observed a significant decline in IMD incidence during the years of the global COVID-19 pandemic, 2020–2022. In 1998–2019, the national IMD incidence rate was 0.9/100,000 population, compared to 0.3/100,000 population in 2020–2022 (0.4/100,000 in 2020 and 0.2/100,000 in 2021 and 2022) (RR = 3.5, 95%CI 2.78–4.46, *p* = 0.0001).

The mean number of IMD cases notified annually countrywide decreased during the COVID-19 pandemic years by 65% compared to the previous years (in the years 2020–2022—the mean number of IMD cases was 24 ± 7 cases annually, compared to 68 ± 16 IMD cases in the years 1998–2019, *p* = 0.001, Figure 1).

### 3.2. Invasive Meningococcal Disease Case Characteristics 

The study group of IMD cases with laboratory results for 2007–2022 included 521 patients, of whom 47 have died (an overall case fatality rate, CFR, 9%, Table 1). The CFR was higher in older IMD cases, 6.9% and 15.4% in cases under 18 years and older than 18 years, respectively (OR = 2.46, 95%CI 1.25–4.74, *p* = 0.004). There was male predominance (60.1%) among the IMD cases, varying with age, 61.3% among cases younger than 45, and 51.7% among those older than 45. The overall mean and median ages of IMD cases were 14 ± 21.8 and 3 years, respectively. The IMD cases were categorized into six age groups: infants aged <1 year, toddlers aged 1–4 years, children aged 5–9 years, adolescents aged 10–17 years, persons aged 18–44 years, and persons aged ≥45 years. Most IMD cases (75%) were younger than 18 years, 59% were younger than 5 years, and 33% were under 1 year. Of the infants aged under 1 year (n = 174), the mean age was 5.1 ± 3 months, and the median age was 4.3 months. Over half (55.3%) of the IMD cases resided in low socio-economic localities (rank of 1–3). About 78% of IMD cases were reported among Jews and about 22% among Arabs. About half of the IMD cases (45.7%) occurred during the winter months of December to March. Serogroup B was the predominant serogroup accounting for 62.6% of the IMD cases. We compared the IMD case characteristics according to the serogroups: serogroup B (n = 326) vs. all other serogroups combined (NON-B) (n = 195). Several variables showed differences between the two above groups. These included the cases’ age (younger in serogroup B cases, median age 1.9 years vs. 11.2 years in NON-B), socio-economic rank (lower in serogroup B cases, median rank 2 vs. 3.5 in NON-B), and the case fatality rate (lower among serogroup B cases, CFR of 7.9% vs. 10.8% in the NON-B cases). Notably, the CFR differed between the *N. meningitidis* serogroups: 57% for serogroup A (4/7 cases), 20.7% for serogroup C (6/29 cases), 12.5% for serogroup Y (10/80 cases), 7.3% for serogroup W135 (3/41 cases), and 5.3% for NG (2/38 cases). In a multiple regression model analysis designed for risk markers for IMD mortality, only the IMD cases’ age (older age group) and socio-economic rank (lower) were associated with IMD mortality.

### 3.3. Distribution of N. meningitidis Serogroups

**Figure 2:**
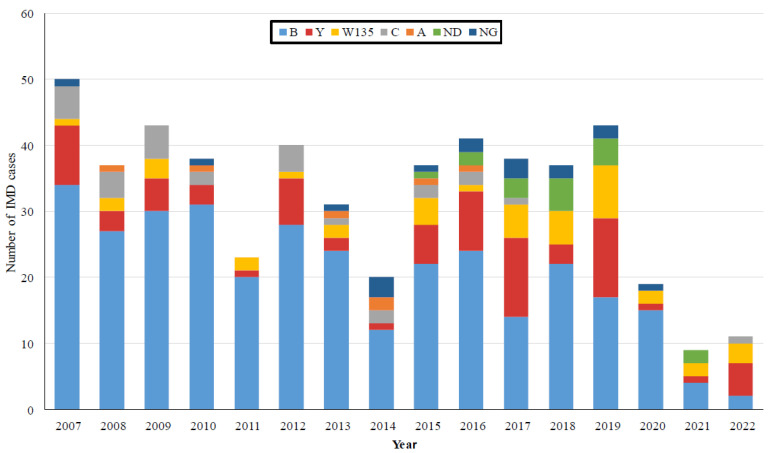
The annual distribution of *N. meningitidis* serogroups in bacterial isolates during the years 2007–2022 in Israel.

Figure 2 presents the distribution of *N. meningitidis* serogroups from isolates received from 2007 to 2022. Overall, the leading serogroup has been *N. meningitidis* B, followed by serogroups Y, W135, C, and A. In 38 cases (7.3%), the *N. meningitidis* isolate was not allocated. During the years 2007–2022, considerable changes have occurred. Along with fluctuations and a trend of decline in the number of IMD cases (Figure 1), several alterations occurred in the distribution of *N. meningitidis* serogroups during the study years (Figure 2). The circulating meningococcal serogroups have altered considerably since the emergence of COVID-19. The relative proportion of *N. meningitidis* serogroup B showed a general decline, most prominently during the COVID-19 pandemic years (2020–2022) when the serogroup B accounted for 53.8% of IMD cases notified nationally, followed by *N. meningitidis* serogroup Y, which accounted for 17.9%. This trend has been even more notable during the years 2021–2022, when *N. meningitidis* serogroup B and serogroup Y each accounted for 30% of IMD cases nationally.

### 3.4. N. meningitidis Serogroups Trends According to Age Groups

**Figure 3:**
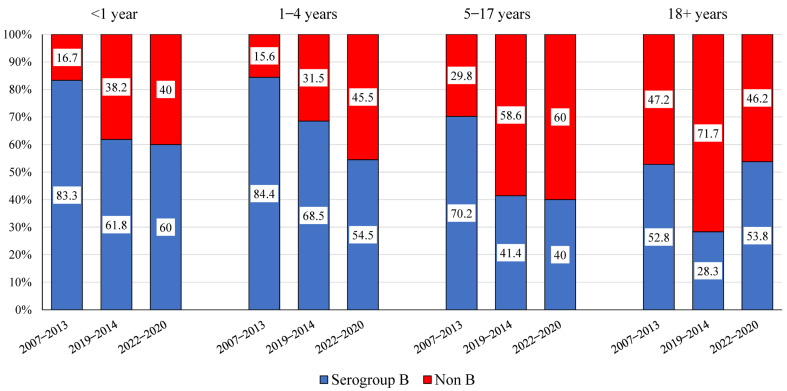
Comparison of *N. meningitidis* serogroups distribution, shown as serogroup B compared to the non-B serogroups in three periods: 2007–2013, 2014–2019, and 2020–2022. IMD cases are presented in four age groups: infants aged <1 year, toddlers aged 1–4 years, children aged 5–17 years, and persons aged 18 years and above.

Figure 3 presents the comparison of *N. meningitidis* serogroup distribution, shown as serogroup B, compared to the assembled group of *N. meningitidis* non-B serogroups. The distribution of *N. meningitidis* serogroups among notified IMD cases, according to four age groups (infants under 1 year, children aged 1–4 years, children aged 5–17 years, and persons aged 18 years and above) is presented for three periods, the years 2007–2013, 2014–2019, and 2020–2022. The distribution of *N. meningitidis* serogroups varied among age groups and over time. Between 2007 and 2013 and 2014 and 2019, the proportion of serogroup B declined in all groups (*p* = 0.001). In children aged 0–17 years, the serogroup B proportion was similar in 2014–2019 and 2020–2022. 

Overall, *N. meningitidis* serogroup B has been predominant in children aged 0–17 (70.6%, 2007–2022) and less prevalent in persons aged 18 years and above (41.3%, 2007–2022, *p* = 0.0001). Among children aged 0–17, the proportion of *N. meningitidis* serogroup B declined with time (80.6% in 2007–2013, 60.3% in 2014–2019, and 53.8% in 2020–2022, *p* = 0.0001). 

### 3.5. Core Genome Multi-Locus Sequence Typing (cgMLST) Analysis

**Figure 4:**
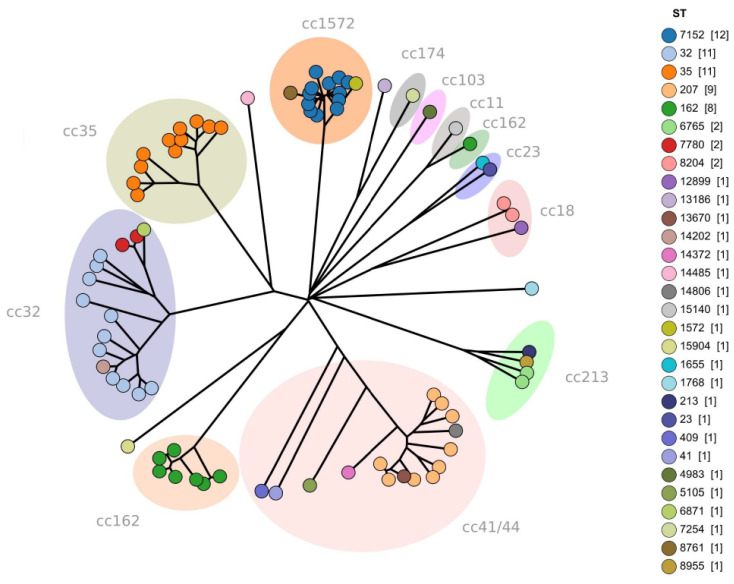
A neighbor-joining diagram of core-genomic multi-locus sequence typing (cgmlst) analysis of serogroup B *N. meningitidis* isolates during the years 2017–2022 in Israel (n = 79 isolates are colored according to sequence type (ST), grouped and labeled according to clonal complex (cc). The phylogenetic tree was constructed with Grapetree, provided by Enterobase v1.1.3. The clonal complex (cc) networks were drawn and edited with the graphical Inkscape tool (www.inkscape.org/en/, accessed on 30 May 2023).

Figure 4 presents a neighbor-joining diagram to show the core-genomic multi-locus sequence typing (cgMLST) analysis of 79 serogroup B *N. meningitides* (MenB) isolated received between 2017 and 2022 in Israel. The phylogenetic tree was constructed with Grapetree, provided by Enterobase v1.1.3. Each circle represents an isolate. Marked texts and colors indicate the clonal complexes (CCs) group. The leading CCs found were 32 (15 strains), 41/44 (the hyper-invasive clonal complexes, 15 strains), and 1572 (14 strains).

### 3.6. Potential Strain Coverage Analysis

A supplementary laboratory data analysis has been performed aiming to evaluate the potential immunity prediction with reference to the two commercially available Meningococcal B vaccines. This specific analysis has been based on a limited number of *N. meningitides* serogroup B isolates (including 72 isolates, starting from 2017, Supplementary Table S1). The prediction analysis outcomes propose an estimated strain coverage for the Meningococcal B vaccines of about 25% for the MenB-4C vaccine (Bexsero) and 48.6% for the MenB-fHbp vaccine (Trumenba). 

### 3.7. Trends in Clonal Complexes (CC)

We compared the prevalence of the leading CCs before and after the COVID-19 pandemic. During 2007–2019, the leading CCs were (listed by frequency): 32, 41/44, 23, 11, 35, 1572, 162, and 213, accounting for 89% of the IMD cases (excluding the NG cases). During 2020–2022 (COVID-19 pandemic years), the leading CCs were (listed by frequency): 23, 11, 41/44, 162, 32, 35, 1572, and 213, accounting for all IMD cases (excluding the NG cases). The leading CC during the years 2007–2022 was 32, which accounted for 24.2% (112/462) of all IMD cases. The most prominent change during the COVID-19 pandemic 2020–2022 years was observed regarding the CC 32 (decline from 25.5% to 8.6%, *p* = 0.014). The CCs showing an increase in fraction during the years 2020–2022 were: CC 23 (from 16.4% to 20%), CC 11 (from 9.1% to 20%, *p* = 0.047), CC 162 (from 4.2% to 11.4%), and CC 213 (from 2.8% to 8.6%). Notably, most of the IMD-related mortality during 2007–2022 was attributed to four clonal complexes: CC 32 (n = 13/47 fatalities, 27.6%), CC 11 (n = 9/47 fatalities, 19.1%), CC 41/44 (n = 6/47 fatalities, 12.8%), and CC 23 (n = 5/47 fatalities, 10.6%). 

## 4. Discussion

During the last two decades, the epidemiology of invasive meningococcal disease has presented considerable fluctuations in many countries, both overall and regarding the *N. meningitidis* serogroups distribution, as well as variation among the cases’ age groups [18,19,20]. Moreover, an overall trend of decline in IMD incidence during the COVID-19 pandemic years has been described in several studies [10,11,12,13]. 

The decline in IMD during the COVID-19 pandemic has been noted in many countries, along with a decline in invasive infections due to other respiratory pathogens, e.g., *Haemophilus influenzae* and *Streptococcus pneumoniae*, yet, a decline has not been observed in *Streptococcus agalactiae*, a non-respiratory pathogen [10]. A recent review including four southern European countries (Italy, Portugal, Greece, and Spain, 1999–2019) displayed marked dynamics in incidence rates of IMD across the years [21]. The reported overall IMD incidence in Europe has declined from 0.95 per 100,000 population in 2008 (4744 cases) to 0.62 per 100,000 in 2017 (3212 cases), a 34% reduction [18]. Correspondingly, our study data showed that, during 1998–2022, the overall incidence rate of IMD in Israel was 0.8/100,000 population, with considerable variability in annual incidence (highest IMD incidence rate in 2005, 1.4/100,000 population and lowest IMD incidence rates in 2021–2022, 0.2/100,000 population). Similar overall IMD incidence rates (about 0.9/100,000 population, ECDC network data) have been reported in European countries [22]. A trend of decline in IMD incidence rates during the COVID-19 pandemic years was also apparent in Israel (0.9/100,000 population during 1998–2019 compared to 0.3/100,000 during 2020–2022). The annual number of notified IMD cases nationally decreased considerably by 65% during 2020–2022 compared to 1998–2019. The bases for this observed decline in IMD incidence might be associated with decreased infection transmission, with reference to the application of various control measures during the COVID-19 pandemic. These measures included lockdowns, school and kindergartens closures, social distancing, and prolonged extensive use of facemasks.

In previous studies in Israel, most IMD cases were reported in young children, mainly in low socio-economic communities (Orthodox Jewish and Arab), with peak incidence rates in infants under 1 year of age [6,7,8,23,24]. In the past two decades in southern European countries, the incidence of IMD was highest in infants and young children [21]. In the current study, most (75%) of the IMD cases notified nationally during 2007–2022 were younger than 18 years, 59% were younger than 5 years, and 33% were infants younger than 1 year. Additionally, over half of the notified IMD cases were residents of low-ranked socio-economic localities. The IMD-associated disease burden is considerable, both globally and in Israel, with mortality and long-term morbidity, including neurological disabilities and functional sequelae [6,7,8,20,23,24,25,26]. The overall IMD case fatality rate in our study population was 9%, within the previously reported range [3]; mortality risk was associated with cases’ age and socio-economic status, similar to previous reports [23,25,26,27]. According to the World Health Organization, meningitis caused an estimated 250,000 deaths in 2019 worldwide, leaving one in five affected individuals with long-term devastating sequelae [4]. The Global Burden of Disease (GBD) Study reported that an estimated 141,000 fatalities (range 96,800–203,000) in 2019 were attributed to *N. meningitidis* infections [26]. It is to be noted that estimates may vary between different disease burden models. In an evaluation of global health models estimating meningitis mortality, the calculated case fatality rate, based on the GBD 2017 estimates, was 8% for meningococcal meningitis [27].

There was a male overrepresentation (60%) among IMD cases, mainly in those aged under 45 years. Compatibly, in a meta-analysis assessing IMD incidence gender differences in 10 countries, the male-to-female incidence rate ratios were significantly higher among males under 45 years and higher among females in age groups over 45 years [28]. Gender-associated variances in IMD incidence should probably be further investigated. 

The distribution of *N. meningitidis* subgroups showed considerable dynamics. The leading serogroup during the years 2007–2022 in Israel has been *N. meningitidis* B (63%), followed by the serogroups Y (15%), W135, C, and A. The distribution of serogroups differed with the IMD cases’ age groups: serogroup B dominated in the younger IMD cases, aged 0–9 years, compared to the cases aged 10 years and above (74.5% and 39%, respectively, 2007–2022). In data from the US, serogroup B accounts for some 60% of IMD cases in infants aged under 1 year, while serogroups C, Y, and W account for some 66% of IMD cases in children older than 11 years [29].

Notably, the proportion of serogroup B *N. meningitidis* of IMD cases in Israel has declined significantly from 75% (during 2007–2013) to 52% (during 2014–2022). Furthermore, during 2021–2022, the *N. meningitidis* serogroups B and Y each accounted for 30% of IMD cases nationally. Comparable trends in the distribution of the *N. meningitidis* serogroups have been described in Europe [18], with the fraction of *N. meningitidis* serogroup B decreasing markedly from 71.5% of all the IMD cases in 2008 to 48% in 2017. Concomitantly, the fractions of *N. meningitidis* serogroups W and Y increased from 1.7% and 3.0% in 2008 to 16.0% and 10.9% in 2017, respectively [18]. Meningococcal disease patterns tend to be cyclic and correlate with circulating *N. meningitidis* serogroups and clonal complex genotypes. Hence, the recent evolving epidemiology of invasive Meningococcal disease attributed to *N. meningitidis* serogroups W, Y, X, E, and nongroupable meningococci has become increasingly important and necesitates further follow-up [30]. 

Data on *N. meningitidis* carriage rates can also support epidemiological evaluations. An assessment of *N. meningitidis* carriage in young Israeli adults revealed sizable overall carriage rates (20.1%) and encapsulated strains (6.7%). Serogroups B (49.2%) and Y (26.7%) were prevalent, C, W, and X-scarce, and group A absent [31]. The most notable clonal complexes (CCs) were CC23, CC32, and CC44/41 [31].

Our data propose that the prevalence of the leading CCs nationally has also shown dynamics during the COVID-19 pandemic years. During 2007–2019, the leading CCs were 32, 41/44, 23, 11, 35, 1572, 162, and 213. During 2020–2022, the leading CCs differed and were 23, 11, 41/44, 162, 32, 35, 1572, and 213. Overall, the leading CC (during 2007–2022) was CC 32, which accounted for about a quarter of IMD cases. During the COVID-19 pandemic years 2020–2022, the CC 32 prevalence showed a marked decline. Notably, the distribution patterns of the *N. meningitidis* clonal complexes correlate with epidemic potential as well as antibiotic resistance [32,33]. A recent report from the fourth Global Meningococcal Initiative summit meeting [33] indicated that some *N. meningitidis* clonal complexes, mainly CC4821 and CC11, show considerable antibiotic resistance (to ciprofloxacin and beta-lactams). The dynamics in clonal complexes distribution warrants ongoing surveillance. 

Invasive meningococcal disease may currently be considered a vaccine-preventable disease [8,29,32,33,34]. Pizza et al. [34] reviewed meningococcal vaccines; many countries have updated the recommendations from monovalent conjugate vaccines to quadrivalent MenACWY conjugate vaccines and protein-based MenB vaccines. Serogroup B vaccines are recommended in risk groups, for outbreak control, and in some countries in the national programs, based on country-specific analysis [35]. However, the modeling of country-specific economic burden and cost-effectiveness evaluation of the available MenB vaccines is complicated [36]. Israel’s recommendations include MenACWY conjugate vaccines (risk groups and travelers) and protein-based MenB vaccines (risk groups mostly immune-compromised and children). The Israel Defense Forces (IDF) had adopted, some two decades ago, a MenACWY vaccination program aimed at army recruits and showing a decline in incidence in the IDF [37,38]. However, in Israel, most IMD cases occur in young children (in the current study, the IMD cases’ median age was three years), and most cases are attributed to serogroup B. Israel’s National Immunization Technical Advisory Group (NITAG) has recommended the use of the MenB vaccine in infants and toddlers in 2019 [39]. To date, the MenB vaccine has not been introduced into the routine (free of charge) childhood vaccination program, mainly due to budgetary constraints [39,40]. The application to include the 4CMENB vaccine into the routine vaccination program has been recently submitted to the national committee for the 2023 expansion of the health services basket and has not been approved due to high costs [41]. The MenB vaccine for children is currently available, with considerable co-payment, in the complimentary insurance programs of the health funds [30,39]. Therefore, it is estimated that MenB vaccination coverage is relatively low (about 20%) and that receipt is mainly in high-income groups, with negligible receipt in low-income groups [41]. Notably, our data show that low socio-economic localities are overrepresented among Israel’s IMD cases, similar to previous studies [7,8]; children in low-income localities are probably less likely to be vaccinated by the co-payment arrangement.

The UK introduced the 4-component, protein-based meningococcal serogroup B vaccine (4CMenB; Bexsero) into the national vaccination program in 2015 [42]. The adjusted vaccine effectiveness against MenB disease was 52.7% (2-dose priming) and 59.1% (2-dose priming plus booster) [42]. The 4CMenB vaccine effectiveness has been estimated in Spain as 76% against invasive meningococcal disease caused by any serogroup [43]. The aim of global control of meningococcal infections requires the development of next-generation vaccines, e.g., the MenACWYX conjugate vaccine and the MenABCWY combined vaccine [34,44,45]. Another major global public health challenge is the vast inequity in access to vaccines and vaccination programs [46].

The main limitations of the current study are associated with the application of secondary use of laboratory data. Considering that the National Reference Center for Meningococci relies on the *N. meningitidis* isolates sent by the clinical, microbiological laboratories, IMD cases in which isolates were unavailable were not included in the database (for the *N. meningitidis* subgroup analysis and the clonal complex analysis). Additionally, the evaluation results might have been affected by the sequential implementation of the progressive laboratory methods.

## 5. Conclusions

In conclusion, the current study offers an assessment of invasive meningococcal disease trends in Israel from 2007 to 2022. The incidence trends of invasive meningococcal disease showed a significant decline during the COVID-19 pandemic (2020–2022). Overall, most *N. meningitidis* isolates were of serogroup B and the most prevalent CC was CC32. Furthermore, the distribution of *N. meningitidis* serogroups and clonal complexes was also altered markedly in recent years. Ongoing comprehensive invasive meningococcal disease surveillance is necessary to assess trends in circulating strains and support health policy decision-making regarding meningococcal vaccination programs in the coming years.

## Figures and Tables

**Table 1:** Case characteristics, invasive meningococcal disease (IMD), 2007–2022.

Variable	IMD Cases (n = 521)
Age (mean ± SD), years	14 ± 21.8
Age median, years (IQR)	3 (IQR = 0.6–18.2 years)
Age group, years	
<1	174 (33.4%)
1–4	133 (25.5%)
5–9	50 (9.6%)
10–17	34 (6.5%)
18–44	67 (12.9%)
≥45	63 (12.1%)
Gender	
male	313 (60.1%)
female	208 (39.9%)
Socio-economic status	
Rank 1–3	288 (55.3%)
Rank 4–6	117 (22.4%)
Rank 7–10	116 (22.3%)
Ethnicity	
Jews and others	406 (77.9%)
Arabs	115 (22.1%)
Seasonality	
December-March	238 (45.7%)
April–July	147 (28.2%)
August–November	136 (26.1%)
Mortality	47 (9%)
Source of bacterial isolation	
Blood culture	331 (63.5%)
CSF	175 (33.6%)
other	15 (2.9%)
Serogroup	
B	326 (62.6%)
Y	80 (15.4%)
W135	41 (7.9%)
C	29 (5.6%)
A	7 (1.3%)
Not allocated	38 (7.3%)

## Data Availability

The epidemiological data that support the findings of this study are not openly available due to reasons of confidentiality.The laboratory data concerning the Neisseria meningitidis bacterial clones may be available from the authors upon reasonable request.

## Supplementary Materials

The following supporting information can be downloaded at: https://www.mdpi.com/article/10.3390/microorganisms11092212/s1, Table S1: MenDeVAR Index Analysis of Protein-Based Meningococcal Vaccines. The table presents the results of the Meningococcal Deduced Vaccine Antigen Reactivity (MenDeVAR) Index analysis, assessing the differential reactivity of two protein-based vaccines against meningococcal pathogens. The data was derived from whole genome sequencing (WGS) of culture-confirmed isolates, providing valuable genomic insights into vaccine antigen responsiveness.
