# Supplementary material for: Invasive Disease Due to Neisseria meningitidis: Surveillance and Trends in Israel Prior to and during the COVID-19 Pandemic
Source: Microorganisms. 2023 Aug 31;11(9):2212. doi: 10.3390/microorganisms11092212 (PMC10537818; doi:10.3390/microorganisms11092212)
Supplement: Supplementary file 1 [file microorganisms-11-02212-s001.zip › microorganisms-2508664-supplementary.pdf]

Serogroup	N=		Coverage by Bexsero					Coverage by Trumenba		
			insufficient data	exact match	cross-reactive	none		insufficient data	exact match	cross-reactive
B	72		53	6	12	1		37	4	31
C	1		1							1
NG	8		7			1		6		2
W	24		4		20			23		1
Y	31		28	1		2		7		24
Total	136		93	7	32	4		73	4	59
